# Supplementary material for: Modelling Skylarks (Alauda arvensis) to Predict Impacts of Changes in Land Management and Policy: Development and Testing of an Agent-Based Model
Source: PLoS One. 2013 Jun 6;8(6):e65803. doi: 10.1371/journal.pone.0065803 (PMC3675089; doi:10.1371/journal.pone.0065803)
Supplement: Supporting Information S4 — The skylark ODdox as a zipped archive. (ZIP) [file pone.0065803.s004.zip › Skylark_ODdox/functions_func_0x70.html]

ALMaSS Skylark ODdox: Class Members - Functions


ALMaSS Skylark ODdox
 2.0


- Main Page
- Related Pages
- Classes
- Files

- Class List
- Class Index
- Class Hierarchy
- Class Members

- All
- Functions
- Variables
- Typedefs

- a
- b
- c
- d
- e
- f
- g
- h
- i
- j
- k
- l
- m
- n
- o
- p
- r
- s
- t
- u
- v
- w
- y
- z
- ~

### - p -

- Parkland()
  : Parkland
- ParseCfgLine()
  : Configurator
- PermanentSetaside()
  : PermanentSetaside
- PermPasture()
  : PermPasture
- PermPastureLowYield()
  : PermPastureLowYield
- PermPastureTussocky()
  : PermPastureTussocky
- Pesticide()
  : Pesticide
- PesticideEvent()
  : PesticideEvent
- PesticideResponse()
  : Skylark\_Base
  , Skylark\_Nestling
  , Skylark\_PreFledgeling
  , Skylark\_Female
  , Skylark\_Male
- PesticideTrialControl()
  : PesticideTrialControl
- PesticideTrialToxicControl()
  : PesticideTrialToxicControl
- PesticideTrialTreatment()
  : PesticideTrialTreatment
- PigsAreOut()
  : Farm
- PigsAreOutForced()
  : Farm
- PigsOut()
  : Farm
- PitDisused()
  : PitDisused
- PolygonDataVector()
  : PolygonDataVector
- PolysDump()
  : Landscape
- PolysRemoveInvalid()
  : Landscape
- PolysValidate()
  : Landscape
- PolytypeToString()
  : Landscape
- Pop()
  : LowPriority< ELEMTYPE >
- Population\_Manager()
  : Population\_Manager
- PositionMap()
  : PositionMap
- PreCachePoly()
  : SkTerritories
- PreEvaluateHabitat()
  : SkTerritories
- PreEvaluateHabitatStripX()
  : SkTerritories
- PreEvaluateHabitatStripY()
  : SkTerritories
- PreEvaluateQualGrid()
  : SkTerritories
- PreFillQualCache()
  : SkTerritories
- PreFillQualGrid()
  : SkTerritories
- PreFillTerrPolyLists()
  : SkTerritories
- PreMakeForIterator()
  : SkTerritories
- PrePoly2Qual()
  : SkTerritories
- PrePolyNQual()
  : SkTerritories
- PreProcessLandscape2()
  : SkTerritories
- Probe()
  : Population\_Manager
  , TSkylarkList< T >
- probe\_data()
  : probe\_data
- ProbeFileInput()
  : Population\_Manager
- ProbePOM()
  : Skylark\_Population\_Manager
- ProbeReport()
  : Population\_Manager
- ProbeReportPOM()
  : Skylark\_Population\_Manager
- ProbeReportTimed()
  : Population\_Manager
- ProductApplication0()
  : Farm
- ProductApplication1()
  : Farm
- Push()
  : LowPriority< ELEMTYPE >
- Put()
  : RasterMap


- Generated on Thu Jan 10 2013 13:15:39 for ALMaSS Skylark ODdox by
   1.8.1.1
